# Perceptions of GLP-1 RA Use for Children With Obesity Among Caregivers With Food Insecurity: A Qualitative Study

**DOI:** 10.1001/jamanetworkopen.2025.52825

**Published:** 2026-01-07

**Authors:** Kathryn M. Stephenson, Naomi R. M. Schwartz, Hannibal Person, Niviann Blondet, Maria E. Benitez-Cortez, Mason Nuding, Stephanie A. Kraft, David L. Suskind

**Affiliations:** 1 Division of Pediatric Gastroenterology & Hepatology, Department of Pediatrics, Seattle Children’s Hospital, Seattle, Washington; 2 Department of Subspecialty Pediatrics, Gastroenterology, Kaiser Permanente Medical Group, Oakland Medical Center, Oakland, California; 3 Research Integration Hub, Seattle Children’s Hospital, Seattle, Washington; 4 Department of Bioethics and Decision Sciences, Geisinger College of Health Sciences, Danville, Pennsylvania

## Abstract

**Importance:**

With the approval of glucagon-like peptide-1 receptor agonists (GLP-1 RAs) for pediatric obesity, families are increasingly considering pharmaceutical treatment alongside lifestyle optimization for their children, although how caregivers will make these decisions remains unexplored. This is especially important in contexts where food insecurity may limit the feasibility of lifestyle change and adjunct therapy could improve outcomes.

**Objective:**

To explore how caregivers make decisions regarding the inclusion of GLP-1 RAs for pediatric obesity treatment in the context of food insecurity.

**Design, Setting, and Participants:**

In this qualitative study, eligible participants were caregivers (aged ≥18 years) of children (aged <18 years) with obesity and metabolic dysfunction–associated steatotic liver disease who screened positive for moderate-severe food insecurity between July 1, 2022, and October 31, 2023. Caregivers were recruited from pediatric gastroenterology clinics of a regional academic health care system. Semistructured interviews via telephone or video were offered in participants’ preferred language between December 1, 2023, and April 30, 2024.

**Main Outcomes and Measures:**

The main outcome was factors influencing caregiver decisions regarding medication use for their child’s metabolic disease. Interview transcripts were translated to English as needed, then thematically analyzed inductively by 2 research investigators to identify themes within an array of caregivers’ decision-making processes.

**Results:**

Of 37 eligible caregivers, 21 completed interviews, 20 of which were included in the analysis. Of the 20 caregiver participants (19 [95%] mothers; mean [SD] age, 40.5 [6.1] years), 18 (90%) were born outside the US, 13 (65%) had not completed high school, and 15 (75%) used a language other than English in clinical settings. Of the 20 child patients, 18 (90%) were male, and mean (SD) age at interview was 12.9 (2.9) years. All caregivers had received lifestyle-related recommendations from their child’s physicians and had been referred to dietetics; 10 (50%) had met with a dietitian. Twelve (60%) desired adjunct pharmacotherapy for their child. Three common themes influenced caregiver decision-making about GLP-1 RA use: (1) prior experience with lifestyle change, (2) trust in the safety and efficacy of GLP-1 RAs, and (3) values regarding optimal care for children. An updated conceptual framework was applied to illustrate caregivers’ decision-making.

**Conclusions and Relevance:**

In this qualitative study, children’s caregivers experiencing food insecurity expressed interest in both lifestyle-only and medication-inclusive approaches, with decisions shaped by lived experience, perceptions of safety, and values. Shared decision-making conversations in clinical settings that acknowledge these influences—and address barriers to both treatment options—could foster more family-centered, equitable, and effective pediatric obesity care.

## Key Points

**Question:**

What factors shape how caregivers experiencing food insecurity perceive the use of glucagon-like peptide-1 receptor agonists (GLP-1 RAs) for children with obesity and metabolic disease?

**Findings:**

In this qualitative study including 20 caregivers, 3 common themes influencing decision-making about GLP-1 RA use for children emerged: quality of prior experiences with lifestyle change, trust in the safety and efficacy of medication, and personal beliefs about caring for children.

**Meaning:**

These findings suggest 3 key entry points for shared decision-making about GLP-1 RA initiation in food-insecure settings: exploring prior barriers to achieving lifestyle change, building understanding around all treatment options, and aligning care with family values.

## Teaser

This qualitative study explores perceptions among caregivers who have food insecurity about use of glucagon-like peptide-1 receptor agonists (GLP-1 RAs) for children with obesity and metabolic disease.

## Introduction

In 2023, the American Academy of Pediatrics added pharmacotherapy alongside lifestyle modification to its management recommendations for obesity in children aged 12 years or older.^1^ This marked a significant expansion of treatment options for a condition that affects nearly 1 in 5 US adolescents.^2^ Addressing childhood obesity is imperative given its associations with morbidity and mortality in adulthood.^3^

Although lifestyle modification remains the first-line approach to addressing obesity, its effectiveness is limited, with particular challenges in low-income populations.^4^ Only 5% to 23% of children have sustained a reduction of at least 5% in body mass index (BMI) over 1 to 3 years using lifestyle modification in randomized clinical trials.^5,6,7,8^ General-population findings reflect similar trends: approximately 60% of childhood obesity carries into adolescence and 80% of adolescent obesity carries into adulthood.^9^ In contrast, combining glucagon-like peptide-1 receptor agonists (GLP-1 RAs) such as semaglutide^8^ with lifestyle education has resulted in 15% BMI reductions in clinical trials, approaching the 20% BMI reduction seen after bariatric surgery in teens.^10^

Beyond clinical efficacy, GLP-1 RAs may offer an opportunity to advance health equity for children facing structural barriers to lifestyle change by providing an accessible adjunct treatment option. Childhood obesity is closely associated with social determinants of health.^11^ Food insecurity—defined as limited or inconsistent access to adequate food—is associated with poor nutrition and unhealthy food environments, such as food deserts and swamps,^12^ that can undermine families’ ability to make lifestyle changes. Clinical and research outcomes in pediatric obesity and metabolic disease are poorer in the context of food insecurity.^13,14,15^

As new pediatric clinical practices surrounding the use of GLP-1 RAs take shape, it is essential to incorporate the voices of families affected by food insecurity. In line with principles of restorative justice, lasting and equitable system-level change must be grounded in the lived experiences of those most impacted. Until now, no studies to our knowledge have examined how families navigating food insecurity approach decisions surrounding GLP-1 RA use for children. This study aimed to explore caregiver decision-making processes regarding GLP-1 RA use for pediatric obesity and metabolic dysfunction–associated steatotic liver disease (MASLD) in the context of food insecurity.

## Methods

This qualitative study was conducted at outpatient pediatric gastroenterology clinics of a regional academic health care network. It was approved by the Seattle Children’s Hospital institutional review board, and the Consolidated Criteria for Reporting Qualitative Research (COREQ) checklist was applied.^16^ All participants provided oral informed consent.

### Participant Selection

**Figure 1.**
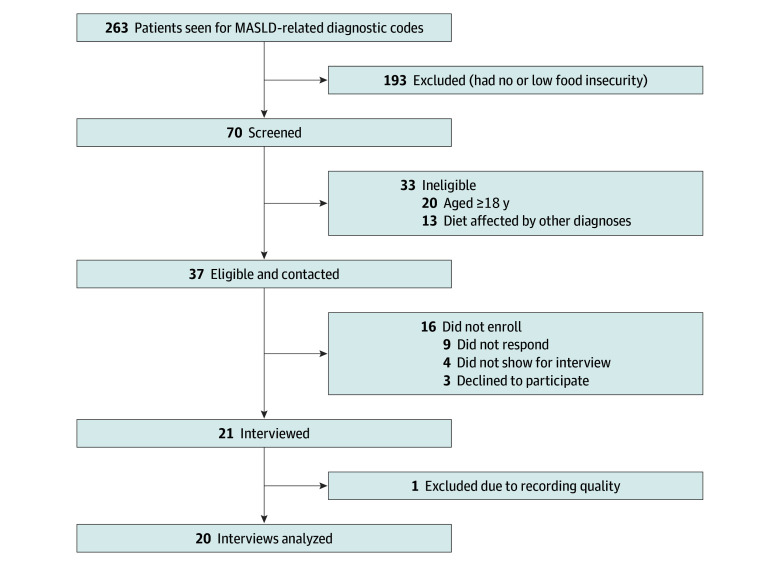
Flow Diagram for Screening and Enrollment of Caregivers MASLD indicates metabolic dysfunction–associated steatotic liver disease.

Caregivers were identified through retrospective review of electronic medical records of pediatric gastroenterology visits from July 1, 2022, to October 31, 2023 (Figure 1). Eligibility required (1) caregiving for a child aged less than 18 years with obesity and MASLD who had attempted at least 2 months of routine physician-recommended dietary changes, (2) moderate to high household food insecurity risk,^17^ and (3) caregiver age of 18 years or older so that caregivers could consent to their own participation. Only caregivers were interviewed, given their responsibility for medical decision-making, food purchasing, and food preparation. To reflect conditions of everyday life, no specific dietary program was required. Families were excluded if the child had a condition affecting dietary intake (eg, tube feeding or eating disorder). All languages were included. Race and ethnicity data, ascertained by family report recorded in the electronic health record, were collected to provide context for understanding potential inequities in families’ experiences and culturally shaped food practices. Race categories were Asian, Black, White, and other (documented as “other” in the electronic health record by the patient’s family) or unknown (documented as “not applicable” by the patient’s family), and ethnicity categories were Hispanic, non-Hispanic, and unknown.

### Interviews and Data Abstraction

Two interviewers, a pediatric gastroenterologist with qualitative research training (K.M.S.) and a research analyst (M.E.B.-C.), conducted 45-minute semistructured interviews between December 1, 2023, and April 30, 2024, via telephone or video. The interview guide, structured around the Capability, Opportunity, Motivation–Behavior (COM-B) framework and Theoretical Domains Framework (TDF) for behavior change,^18^ explored caregivers’ experiences with lifestyle change and interest in GLP-1 RAs at the time of both diagnosis and interview. These frameworks ensured that questions probed multiple domains of behavior, including knowledge surrounding treatment (psychological capability), access to healthy food (physical opportunity), and motivation for sustaining lifestyle change (reflective or automatic motivation) (eAppendix in Supplement 1). Caregivers also completed the Brief Health Literacy Screen^19^ and a short demographic survey. Interviews in languages other than English or Spanish were assisted by a Health Insurance Portability and Accountability Act (HIPAA)–compliant interpreter service. Participants received a $50 gift card and were referred to services where needs where identified.

Baseline clinical and demographic information, including race and ethnicity categorization, was manually abstracted from the electronic health record. Area Deprivation Index^20^ score was determined from each participant’s household address. We defined diet response using alanine aminotransferase (ALT) as a biochemical marker of disease activity: remission (ALT level <38 U/L), response (ALT level >38 U/L but ≤50% of baseline ALT level at diagnosis), and no response (ALT level >38 U/L and >50% of baseline level) (to convert ALT to µkat/L, multiply by 0.0167). Health care coverage for GLP-1 RAs at the time of the interview was determined by verification of each plan’s GLP-1 RA coverage for pediatric obesity.

### Statistical Analysis

Audio and video recordings were transcribed and translated by a HIPAA-compliant service. A pediatric gastroenterologist with qualitative research training (K.M.S.) and a research analyst (N.R.M.S.) conducted a reflexive thematic analysis following Braun and Clarke’s approach.^21^ This allowed themes to emerge inductively from caregivers’ narratives rather than testing predefined hypotheses. Transcripts were reviewed for familiarity, and a preliminary codebook was developed and piloted on 7 transcripts. The finalized codebook was applied to the remaining 13 transcriptions. Coding discrepancies were resolved through discussion with a third analyst (S.A.K.) available for adjudication. Themes were generated through an iterative process of grouping codes into subthemes, identifying patterned meaning across transcripts, and refining for coherence and distinction. Dedoose software, version 9.6.006 (Sociocultural Research Consultants, LLC), facilitated systematic coding and visual inspection of code co-occurrence to support theme development. Data saturation—defined as the point when no new codes or subthemes emerged from successive transcripts—was reached after 16 interviews. The final 4 interviews confirmed existing themes without generating additional codes. Final themes were reviewed against the coded dataset and reapplied for validation.

## Results

All eligible caregivers (n = 37) were invited to participate in the study; 16 did not enroll (9 had no contact and 7 declined or did not attend the interview). Twenty-one completed interviews, with 1 interview excluded due to poor recording quality (Figure 1).

### Characteristics of Respondents

All included caregivers (n = 20) reported experiencing moderate-severe food insecurity (Table). Nineteen interviews (95%) were conducted with mothers and 1 (5%) with a mother and father together; mean (SD) caregiver age was 40.5 (6.1) years. Most caregivers were born outside the US (18 [90%]); used a language other than English for medical care (15 [75%]), specifically Spanish, Vietnamese, or Pashto; and had not completed high school (13 [65%]). Most (14 [70%]) self-reported the lowest health literacy level (“limited”). The caregivers’ households included a median (range) of 2.5 (1-5) children, and 10 households (50%) had 1 caregiver. The child patients (n = 20) were predominately male (18 [90%]; 2 [20%] were female), with mean (SD) age of 12.9 (2.9) years at the time of interview. Five children (65%) were Asian, none were Black, 5 (25%) were White, and 10 (50%) had other or unknown race; 13 (65%) were Hispanic, 6 (30%) were non-Hispanic, and 1 (5%) had unknown ethnicity. Sixteen children (80%) were insured through Medicaid.

**Table.** Demographic and Clinical Characteristics of Participant Families at the Time of Caregiver Interview

Characteristic	Interviews, No. (%)		
	Overall (N = 20)	Interested in pharmacotherapy (n = 12)	Prefer lifestyle alone (n = 8)
Child’s age, mean (SD), y			
At interview	12.9 (2.9)	13.5 (2.5)	12.3 (3.1)
At diagnosis	11.2 (3.1)	12.1 (3.3)	9.4 (4.6)
Child’s sex			
Female	2 (10)	1 (8)	1 (13)
Male	18 (90)	11 (92)	7 (87)
Child’s race			
Asian	5 (25)	3 (25)	2 (25)
Black	0	0	0
White	5 (25)	2 (17)	3 (38)
Other or unknown^a^	10 (50)	7 (58)	3 (38)
Child’s ethnicity			
Hispanic	13 (65)	7 (58)	6 (75)
Non-Hispanic	6 (30)	4 (33)	2 (25)
Unknown	1 (5)	1 (8)	0
Child’s insurance type			
Private	4 (20)	3 (25)	1 (12)
Medicaid	16 (80)	9 (75)	7 (88)
Caregiver born in US	2 (10)	2 (17)	0
State Area Deprivation Index, mean (SD)^b^	5.7 (3.0)	5.7 (3.1)	6.0 (3.1)
Caregiver’s educational level			
<High school	13 (65)	7 (58)	6 (75)
High school diploma	3 (15)	2 (17)	1 (13)
Some college or degree	4 (20)	3 (25)	1 (13)
Caregiver participants			
Mother	19 (95)	12 (100)	7 (88)
Mother and father together	1 (5)	0	1 (13)
Subjective response to diet at interview^c^			
Yes	14 (70)	8 (67)	6 (75)
No	5 (25)	3 (25)	2 (25)
Unknown	1 (5)	1 (8)	0
Objective response to diet at interview^d^			
Remission	3 (15)	1 (8)	2 (25)
Response	8 (40)	3 (25)	5 (63)
No response	8(40)	8 (66)	0
Unknown	1 (5)	0	1 (13)
Language of care			
English	5 (25)	5 (42)	0
Spanish, Vietnamese, or Pashto	15 (75)	7 (58)	8 (100)
Caregiver health literacy^e^			
Adequate	5 (25)	4 (33)	1 (13)
Marginal	1 (5)	0	1 (13)
Limited	14 (70)	8 (67)	6 (75)
RD visits, No.			
0	10 (50)	5 (42)	5 (63)
1-2	7 (35)	4 (33)	3 (38)
≥3	3 (15)	3 (25)	0
Metabolic syndrome at diagnosis	14 (70)	8 (67)	6 (75)
BMI			
<95th percentile	3 (15)	1 (8)	2 (25)
≥95th percentile to <120% of 95th percentile	7 (35)	5 (42)	2 (25)
≥120% to <140% of 95th percentile	4 (20)	1 (8)	3 (38)
≥140% of 95th percentile	6 (30)	5 (42)	1 (13)
Caregivers in primary household, No.			
1	10 (50)	8 (70)	2 (25)
2	10 (50)	4 (30)	6 (75)
Caregiver employment status			
Full time	7 (35)	4 (33)	3 (38)
Part time	0	0	0
Out of work	4 (20)	3 (25)	1 (13)
Stay-at-home parent	9 (45)	5 (42)	4 (50)

Abbreviations: BMI, body mass index; RD, registered dietitian.

^a^ “Other” was documented in the electronic health record by the patient’s family. “Unknown” was documented as NA (not applicable) in the electronic health record by the patient’s family.

^b^ Higher values (on a 10-point scale) indicate a greater index of deprivation (ie, income, educational level, employment, and housing quality) when compared statewide.

^c^ Response to the interview question, “Do you feel like the dietary changes your child has made have been effective for their health?”

^d^ Remission was indicated by alanine aminotransferase level less than 38 U/L; response, by alanine aminotransferase level of 50% of baseline level or less; and no response, by alanine aminotransferase level greater than 50% of baseline level. To convert to µkat/L, multiply by 0.0167.

^e^ Assessed using the BRIEF Health Literacy Screening Tool.

As ascertained from interviews, children had engaged in healthy lifestyle change for a median (range) of 1.7 (0.7-5.7) years. One child (5%) had lost more than 5% of baseline BMI. Eight (40%) showed biochemical response and 3 (15%) achieved biochemical remission. All families had received lifestyle recommendations from a physician and referrals to dietetics; 10 (50%) had met with a dietitian. None had tried medication or participated in intensive health behavior and lifestyle interventions. Twelve children (60%) met current US Food and Drug Administration criteria for use of semaglutide at the time of interview, though none had insurance coverage for the medication. Twelve caregivers (60%) expressed interest in adding pharmacotherapy, while the remaining 8 (40%) reported preferring to continue lifestyle change alone.

### Caregivers’ Decision-Making Processes

Caregivers’ decisions regarding medication use for their child’s metabolic disease were influenced by 3 common themes (Box). These were (1) prior experience with lifestyle change in food insecurity, (2) trust surrounding medication, and (3) beliefs surrounding the optimal care of children.

Box. **Themes, Subthemes, and Representative Quotations From Caregivers About Considering Glucagon-Like Peptide-1 Receptor Agonists for Children**

**Theme 1: Caregivers’ Prior Experience With Lifestyle Change in Food Insecurity**

**Perceived Effects**

“...he looks healthier and he has been able to sleep more, his skin look lighter, and he also said that he feel much better than before. I also said that before, after each meal, he would feel sleepy and he often feel tired at school, but now all that’s gone.” (Participant 34; interview language, Vietnamese)
“They just, they just keep saying us, ‘healthy diet’ and he has a healthy diet, but nothing changed.” (Participant 1; interview language, English)
“He lost 14 pounds, but then gained them back... And that did stress me out, because I said, ‘Everything we did for nothing, right?’ He gained the weight back again.” (Participant 10; interview language, Spanish)
“He tells me, ‘Look, Mom.’ He is surprised when he sees his pictures. He is surprised when he sees his clothes, which now fit him. Now I feel that he likes his change, that is, I’m sure of what I did, because I see it in him.” (Participant 4; interview language, Spanish)
“…he doesn’t like to eat the food and becomes very sad. There’s been days where he doesn’t even eat for the whole day because there’s no things that he likes. But sometimes I go I try to make him happy. At the end of the day, you got to understand he’s still a kid.” (Participant 33; interview language, Pashto)
“I realized that to make that change, it had to be the whole family, not just him. Because if we hurt him, it could happen to my other children, right?” (Participant 4; interview language, Spanish)

**Perceived Feasibility**

“He understands, but he’s very stubborn. So, it’s just really hard. Sometimes I just have to walk away from him. So, it’s a constant battle with him.” (Participant 2; interview language, English)
“We kind of feel sorry for him and give him his soft drink. I think that is where we also fail as parents.” (Participant 10; interview language, Spanish)
“He’s very shy. He likes the food, but he says that at school they give him a tray and he’s embarrassed to tell them what he wants to eat healthy.” (Participant 24; interview language, Spanish)
“Sometimes he cries, and he told, ‘Why I cannot be eating anything? My friend, my friend they eat anything at school. They can eat chips, but you never give me chips. I can’t, I can’t have chips at the school.’ He is sad. He is, he is upset. He is not like other kids. He cannot enjoy like other kids.” (Participant 1; interview language, English)
“I have to go shopping to like 3, 4 different stores just to get the items that I need because of how much they cost in each different store, trying to bargain shop, when there’s bargains in other areas and other stores, not to mention, like I said, having to find those bargains. And then I had to buy a freezer to like deep freeze the meats that are on sale. I go through this whole process. It’s exhausting.” (Participant 22; interview language, English)

**Theme 2: Caregivers’ Trust in Medication**

**Efficacy and Safety of Medication**

“With the diet, it’s very slow. But medication, it’s faster.” (Participant 11; interview language, English)
“I would like to have a medicine that would help him, then he will see the results: ‘Oh, yeah, I am improving.’ Because the diet is very, very slow that he won’t feel anything. And then he used to tell me, ‘What is the use? There is nothing. Nothing is happening.’ But when we go to the next visit and he sees with the diet and with the medication, he is improving, then, ‘Oh, yeah, that is good.’” (Participant 11; interview language, English)
“Well, for me, if they gave him medication or something that would help him burn fat, something that could help him lose weight or help his metabolism speed up, or something that helps him, right? Because as I tell you, he can, but he just stops and gains weight again. Something to help him not to be attracted to food. I don’t know, something to help him.” (Participant 10; interview language, Spanish)
“What I think is that medications always have side effects. So, if we can control our food, control by doing food and the lifestyle, it’s probably better.” (Participant 28; interview language, Vietnamese)
“With the medicine, later on it will turn into cancer. Yes, because, you get sudden relief from eating and is only for someone don’t want to have a healthy diet change. If they don’t want to have a diet change, they will not live long. For me, fresh only, you would live longer. That’s what I think.” (Participant 34; interview language, Vietnamese)
“It is also bad because you take medicine and suddenly you lose weight and suddenly you stop taking the medicine and you gain weight again and even worse, you can get sick from many diseases.” (Participant 20; interview language, Spanish)
“I believe with that medication his healthy habits wouldn’t be very permanent.” (Participant 10; interview language, Spanish)

**Others’ Experiences With Medication**

“I’ve always been overweight, all my life. I’ve lost weight, I gained weight. I’ve been on phentermine. It has worked for me.” (Participant 2; interview language, English)
“I bought the pills and I lost 5 pounds, but when I stopped taking the medication, I gained 10 pounds. So, I don’t recommend that.” (Participant 24; interview language, Spanish)
“…because of the fact of past experiences with family members taking medicine, he’s more of a natural, ‘I’m going to do it without medication’ type.” (Participant 27; interview language, English)
“And I asked the doctor if she could give me medicine; she says, ‘No, because medicine isn’t necessary for that, what’s necessary is the diet. Diet and exercise, and you’ll see results.’” (Participant 24; interview language, Spanish)
“I don’t know what [my daughter] thinks, but I think she’d listen to the doctor.” (Participant 28; interview language, Vietnamese)

**Theme 3: Caregivers’ Beliefs Surrounding Optimal Care of Children**

**Physical Health**

“Since he’s always been chubby, he’s always been overweight, so if it were something beneficial for him and would prevent some diseases, I agree that he should take them.” (Participant 36; interview language, Spanish)
“I think the diet and exercise are very good because it’s something natural. You could say that one puts in effort and that works. On the other hand, if they give you medicine, then you say, ‘I’m going to eat everything anyway since I’m taking medicine.’ It’s not like you’re putting in effort and saying, ‘I’m going to do this to get better.’ And you’re not taking medicine but rather doing something healthy for your own life.” (Participant 24; interview language, Spanish)
“I’m more of a natural approach, versus medicine. Yeah, I mean, holistically, that’s what we aim for.” (Participant 22; interview language, English)

**Socioemotional Well-Being**

“With [patient], I felt like it was hard. I feel like it is hard still, because a dietary change, it isn’t a fast thing. It’s something you have to consistently work on. So, I feel like it was harder because it’s so much easier when someone says, for example, ‘Oh, you have diabetes? Here is metformin. Take metformin and make a life change, make changes and you’ll be okay.’ Compared to, ‘You have to completely do a 360 with diet, that way things can get better.’” (Participant 2; interview language, English)
“I would put the diet gradually. Because it’s hard to put him on a diet right away, like, oh, you cannot eat this anymore. And with the same time, I would like to have a medicine that would help him; then he will see the results: ‘Yeah, I am improving.’” (Participant 11; interview language, English)
“I tried to make him the first time, put everything of me in it and try to keep him on diet, but he’s too young. It’s, like, very hard for him to explain his diagnosis and tell him that you want to eat that stuff and not to eat the other stuff. Because telling that to a kid is very hard for him to understand his situation.” (Participant 33; interview language, Pashto)
“But for me, for now, he’s young and I don’t want him to take medicine for something that we can just, like, change it and do it as a whole. So, no.” (Participant 31; interview language, Vietnamese)

#### Prior Experience With Lifestyle Change in Food Insecurity

Caregivers’ decisions surrounding GLP-1 RA use were largely shaped by prior efforts to implement physician-recommended lifestyle changes (Box). Within this theme, we identified 2 subthemes: (1) perceived effects of lifestyle changes and (2) perceived feasibility of implementing changes in the context of food insecurity.

With regard to perceived effects of lifestyle changes, caregivers who saw meaningful benefits—such as improved appearance, energy, self-confidence, and laboratory values—were often motivated to continue a lifestyle-only approach (Box). When budgets allowed, families who adopted fresh-food diets together saw shared improvements and expressed pride in holistic and/or self-disciplined approaches. For these caregivers, medication sometimes felt like a shortcut that might undercut their child’s agency and achievement. Conversely, caregivers who saw little or no improvement noted persistent weight gain, stagnant laboratory test results, fatigue, and worsening mental health (eg, anxiety or isolation). These experiences led some to question whether dietary advice was effective or even realistic for families reliant on food stamps or pantries. For many, GLP-1 RAs offered a promising way to improve outcomes independent of financial means.

The perceived feasibility of making recommended changes, regardless of health outcomes, also shaped interest in pharmacotherapy (Box). Caregivers who found recommendations manageable tended to desire adjunctive pharmacotherapy less frequently. They identified supports such as culturally appropriate foods at pantries, simplified meal preparation routines, and help from peers or family who share their food culture. Aligning the child’s meals with the family’s reduced burdens of meal preparation promoted modeling and eased emotional strain. Additionally, caregivers who viewed their children as self-motivated or responsible were generally less interested in medication.

Many other caregivers, however, faced significant barriers—stressful meal planning, high costs, and high time investment—with a significant emotional toll. Caregivers described outbursts of overwhelm (loss of emotional control, such as crying or yelling, caused by feeling overloaded) and strong emotional appeals to motivate their children, which affected the whole household. Common challenges included (1) parent-child conflicts, (2) school and social challenges, and (3) cost barriers.

Caregivers noted parent-child conflicts when children perceived delayed benefits of dietary modifications and resentment surrounding food restrictions. This led to resistance and emotional outbursts, particularly when children perceived having different restrictions than their siblings. Caregivers often offered food to maintain cooperation or normalcy, though this provoked guilt and worry. Medication was sometimes seen as an opportunity to accelerate progress and boost motivation.

School and social challenges, such as peer pressure, school cafeteria food, and social eating, also complicated adherence. Hot lunch at school, though less healthy, was cheaper and allowed children to feel included with peers. Some caregivers viewed medication as a tool to support health-related goals without risking social exclusion. They preferred deferring lifestyle change to a more developmentally appropriate time when their child became more socially independent.

Cost barriers were another challenge. High prices, fresh-food spoilage, and time-consuming preparation strained budgets and schedules. Families reported visiting multiple stores to find enough affordable food. To some, medication’s greatest promise was its practicality and sustainability.

#### Trust in Medication

Caregivers’ decisions about medication were also shaped by their degree of trust in medication for pediatric use. Subthemes included perceptions of medication’s efficacy and safety and others’ experiences. These views intertwined with the realities of limited resources (Box).

Concerns about the safety of medications were common, particularly given their novelty and media attention. Parents feared that there may be unknown harms of chronic use, such as cancer. Some questioned whether medication only suppresses visible signs of disease, “band-aiding” the true disease, including the underlying metabolic dysfunction and obesity-related risks. However, the calculus changed where lifestyle change did not feel feasible. Barriers included unaffordable costs of fresh foods, reliance on food pantries with limited options, and time constraints related to work and caregiving. Medication was viewed as a realistic and sustainable tool that some caregivers trusted would be more likely than lifestyle alone to help their child. GLP-1 RAs were considered safer than diets that felt restrictive, ineffective, or financially out of reach.

Trust in pharmacotherapy was also shaped by personal or secondhand experiences with weight-related medications. Anecdotes, such as 1 caregiver’s negative experience with weight rebound due to medications for obesity, influenced reluctance to use similar treatments in children. Conversely, anecdotal and media stories of success with such medications carried considerable weight in favor of medication use. Pediatricians and gastroenterologists played a trusted advisory role in exploring trust surrounding treatment options, though of note, no caregivers were offered pharmacotherapy by a health care practitioner.

#### Beliefs Surrounding Optimal Care of Children

Caregivers’ decisions managing their child’s diagnosis were also shaped by notions of wellness and care responsibility. They often prioritized either their child’s physical health or socioemotional well-being in their approach (Box).

Among the caregivers who discussed the prioritization of physical health outcomes, most expressed fear of the long-term sequelae of obesity. Many expressed guilt that they had not prevented pediatric obesity and/or MASLD and were motivated to make significant sacrifices for their child’s well-being. While caregivers who discussed physical health outcomes tended to favor an exclusive lifestyle approach, some hoped medication would maximize outcomes beyond what lifestyle alone might achieve.

Among caregivers who discussed their child’s socioemotional well-being, balancing dietary restrictions with the child’s sense of happiness and belonging was important. As illustrated in the first theme (prior experience with lifestyle change), enforcing dietary and behavioral controls can be emotionally taxing, leading to guilt or conflict between maintaining dietary discipline and expressing affection. Food insecurity intensified this conflict, as caregivers often felt torn between stretching limited resources to meet dietary recommendations and preserving their child’s sense of normalcy. In this context, medication was sometimes seen as a way to relieve financial and emotional strain. For these caregivers, medication was hoped to promote both the physical and emotional needs of their children.

## Discussion

Our findings underscore the complexity of caregiver decision-making in considering GLP-1 RAs in addition to lifestyle therapy in managing pediatric metabolic disease in the context of food insecurity. Caregivers’ decisions were shaped by a balancing of previous experiences with lifestyle interventions, trust in and understanding of GLP-1 RAs, and deeply held beliefs about what constitutes optimal care for their children. As caregivers navigated treatment options, they weighed both physical health and socioemotional well-being, often considering the broader impact on family quality of life. Notably, the caregivers were fairly equally split in their interest in pharmacotherapy, highlighting the diverse cultural, emotional, and socioeconomic meanings people attach to food and medicine.

Decisional conflict—defined as “personal uncertainty about which course of action to take when choice among competing options involves risk, regret, or challenge to personal life values”^22,23^—commonly arises in multifaceted clinical decisions. A framework elucidating this was offered in previous research on decisional conflict that arises within caregivers considering enteral feeding for children with neurologic impairments.^24,25^ In the context of pediatric obesity, similar core questions arise: What meanings do caregivers ascribe to feeding children? How is quality of life defined for children and caregivers? What factors shape decision-making? This framework emphasizes 3 key influences: (1) context, (2) values, and (3) decision-making processes.

**Figure 2.**
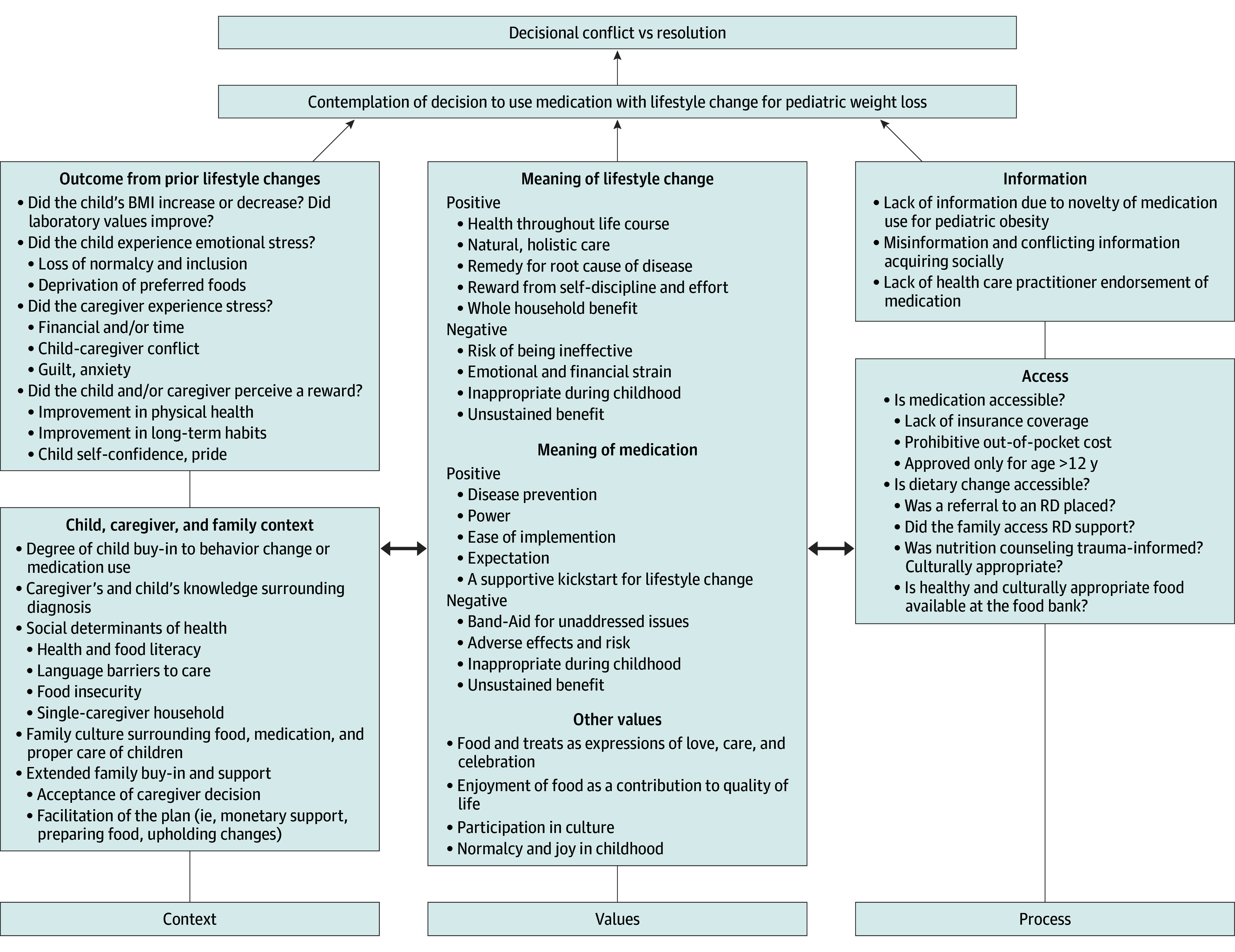
Conceptual Model of Decisional Conflict in Caregiver Decisions Surrounding Pharmacotherapy or Lifestyle-Only Management Adapted from Mahant et al^24^ and Adams et al.^25^ BMI indicates body mass index; RD, registered dietitian.

Applying this framework to our findings provides insights that can improve treatment discussions for metabolic disease (Figure 2). First, shared-decision making should address contextual factors (ie, the characteristics of and circumstances surrounding the family).^25^ Our study identified significant barriers to successful lifestyle interventions (theme 1), including children’s food preferences and social pressures, family customs, cultural norms, and social determinants of health (eg, food insecurity, financial constraints). Routine in-office screening for food insecurity and adverse childhood experiences is important to ensure targeted support and mitigate these barriers.^26,27^

Second, these conversations must recognize the value-laden nature of both lifestyle and pharmacotherapy approaches, as they are closely tied to “attitudes, beliefs, and belief systems.”^25^ In our study, caregivers associated lifestyle changes with positive attributes like holistic care and self-discipline but also perceived them as ineffective or burdensome (theme 1). Adjunctive medication use evoked mixed reactions, ranging from perceptions of efficacy, power, and rapidity to concerns about safety and inefficacy (themes 2 and 3). Respecting caregivers’ values through empathetic dialogue and decision aids—tools that provide clear information while facilitating values clarification—can empower families to make choices aligned with their priorities.^28^

In addition, the decision-making process (ie, “the perceived manner of decision making in concert with health teams”^25^) influences families’ trust in and access to treatment options. Physicians serve as trusted sources of information in clinical decision-making.^29^ However, a gap in health care practitioner support for pharmacotherapy emerged in our study—despite 60% of patients meeting eligibility criteria for pharmacotherapy, no families were encouraged to consider this approach. We suspect this current reticence is multifactorial. A recent qualitative study similarly found only one-fourth of primary care practitioners were willing to prescribe weight loss medication to adolescents, citing resource constraints, time limitations, and doubts about patient adherence or duration of access.^30^ Moreover, none of our participants’ insurance plans would cover the cost of these medications, contributing to practitioner reluctance to recommend them. Given the significant cost of GLP-1 RAs^31^ and limited insurance coverage, there is urgent concern about pharmacoequity.^32^

### Limitations

This study has limitations. As with all qualitative research, our findings are based on self-reported data and may be subject to reporting biases. Social desirability bias may have influenced responses; for instance, some families may have downplayed interest in GLP-1 RA therapy if they perceived it as financially unattainable. In addition, projection bias may have shaped parents’ accounts, as their own experiences with weight loss or medication—though not recorded in this study—could influence how they interpret both their children’s and their own preferences for treatment. This study was not designed to establish associations between variables or to predict trends at the population level. Generalizability is also limited by the study’s focus: we sought to center on the decision-making processes of a small group at high risk for poor obesity care outcomes—caregivers of children with both obesity and metabolic disease experiencing food insecurity of moderate-high risk. While this amplifies a valuable perspective, opinions and needs may differ among larger groups—food-secure families, pediatric patients themselves, and patients managed in primary care settings rather than subspecialty care. Additionally, interviews were conducted during an early phase of GLP-1 RA availability for pediatric obesity. Caregivers’ decision-making processes may evolve as access increases and clinical and public GLP-1 RA experience grows. Future research should explore how these views change over time and across broader populations.

## Conclusions

This qualitative study of caregivers navigating pediatric obesity and metabolic disease in the context of food insecurity offers rich insights into decision-making processes surrounding GLP-1 RA use. Participants’ decisions reflected an interplay of past experiences with lifestyle change, trust in medication safety and efficacy, and deeply held beliefs about caring for children. When families felt tension between these factors, decisional conflict emerged. As GLP-1 RA medications become increasingly accessible, resolving decisional conflict will require attention to each family’s lived experience, education to allow fully informed decisions, and exploration of family values. A shared decision-making model and clinical education tools that honor these complexities are essential for delivering care that is both effective and equitable and should be the focus of future research.

## Notes

Supplement 1. **eAppendix.** Semi-Structured Interview Guide

Supplement 2.
Data Sharing Statement
