# Supplementary material for: Perceptions of GLP-1 RA Use for Children With Obesity Among Caregivers With Food Insecurity: A Qualitative Study
Source: JAMA Netw Open. 2026 Jan 7;9(1):e2552825. doi: 10.1001/jamanetworkopen.2025.52825 (PMC12780928; doi:10.1001/jamanetworkopen.2025.52825)
Supplement: Supplement 1. — eAppendix. Semi-Structured Interview Guide [file jamanetwopen-e2552825-s001.pdf]

Supplementary Online Content

Stephenson KM, Schwartz NRM, Person H, et al. Perceptions of GLP-1 RA use for children with obesity among caregivers with food insecurity: a qualitative study. *JAMA Netw Open*. 2026;9(1):e2552825. doi:10.1001/jamanetworkopen.2025.52825

eAppendix. Semi-Structured Interview Guide

This supplementary material has been provided by the authors to give readers additional information about their work.

© 2026 Stephenson KM et al. *JAMA Network Open*.

eAppendix. Semi-Structured Interview Guide

I am a [researcher/physician] in the GI department at Seattle Children's. I am calling to complete the interview about dietary change for kids scheduled for this time. Is now still a good time to talk?

First, I want to see whether you have any further questions. Did you receive the information sheet about the study in your email? Do you have any new questions? Do I have your consent to include you in our study?

In the next hour, we will talk about [*patient*]'s diet before their diagnosis, their transition to the diet and their experience following the diet over time. Afterwards we will complete a few short questionnaires about your family. You are welcome to decline to answer any questions you prefer not to answer. This will be recorded so that we can analyze it with interviews of other families. When we are finished, Dr. Stephenson will send your $50 gift card for participating in the study to the email address that you provided.

Is it alright with you if I begin recording?

A. Background Preferences (3 min):
(when diagnosed, when started restricting, and what has been restricted?)

1. To begin, please tell me a little bit about your child's diet before they were diagnosed with [*diagnosis*]. Can you walk me through a regular day of what [*patient*] would eat starting at breakfast?
   Prompts:
   - Staples, favorites, special occasion foods
   - Restrictions?
   - What was their meal pattern like? Ex. Food rituals, family meals, school time, snacks
   - Tell me about times when they enjoyed food the most? Ex. Family time, reward, celebration
   - Tell me about the overall balance of their diet.

B. Early Experience with Diet/Lifestyle change (15 min):

1. Now let's shift to your child's transition to the [*lifestyle change*]. Tell me the story of what it was like to learn about [*patient*]'s diagnosis of [*dx*].
   Prompts:
   - Were you familiar with [*diagnosis*]? How would you explain to someone else what this diagnosis means?
   - Were you familiar with the role of food in [*diagnosis*]? Tell me in your own words.
   - Some people find lifestyle change daunting; others find the option a relief. Tell me what it was like for you to learn that the treatment would require a lifestyle change?

© 2026 Stephenson KM et al. *JAMA Network Open*.

2. In your own words, tell me more specifics about the [diet] that your child is on. What rules guide the diet? What foods have you eliminated, or added?

3. How did you learn about the diet? Did you speak with a dietician? Tell me about your conversation. Where else did you find information (friends, internet, family)?

4. How did the first week on the diet go for [*patient*]? The first month?
    - Thinking about all the people in [*patient*]'s life, what was the adjustment like for you? How did the rest of the family at home and the extended family react? How about [*patient*]'s friends? Your friends?
    - Thinking about the major places where [*patient*] spends time, were there any adjustments that you needed to make at home? At school? At special occasions?
5. How difficult do you think changing lifestyle was for [*patient*]?
6. Thinking back to diagnosis, you had had an option to start [*patient*] on a medication to help, would you have chosen this? The medications I am thinking about are the ones you may have heard in the news called GLP-1 receptor agonists with names like semaglutide, Ozempic, wegozy.
    a. Tell me more – open ended

C. Maintenance Experience (30 min):

Thank you for sharing about your family's adjustments. I really appreciate how much insight you've given us into what it has been like. Let's shift to talking about how following the changes are going now.

Capability (knowledge/perception, self-efficacy/control): 10 min

1. First, please tell me more about how you plan meals and buy food for your child. What influences your decisions?

2. Have you gained any new skills or knowledge while adjusting to the new diet?

3. How confident are you that your child is following the diet most of the time? How much personal control do you feel over their food choices?

4. Do you feel like the diet is effective for their [*disease*]---what changes have you noticed in their health? Do you feel like the diet would still be a nutritious choice for someone without their disease?

Opportunity (physical environment, social environment/norms): 10 min

1. (Earlier, you mentioned grocery shopping…) Can you tell me more about how the new diet has affected the cost of the groceries?

2. How has the new diet affected the time it takes to plan and prepare meals?

© 2026 Stephenson KM et al. *JAMA Network Open*.

3. What adjustments have you made in home, school and after school routines to accommodate the new diet?

4. Did changing the diet cause any problems or stress within the family? If so, was it minor, moderate, or major stress? Why?

5. Tell me about how other people, like friends, teachers or other family members support **your child** in following the new diet. What is most helpful? What is challenging?

6. Tell me about how other people, like friends, teachers or other family members support **you** in following the new diet. What is most helpful? What is challenging?

Motivation (beliefs, perceived barriers, intentions, habits, values): 10 min

1. Tell me about how you have talked to your child about following the [rx *diet*] when you are not with them.
   Prompts:
   - What makes following the diet important?
   - How should they explain their diet to others?

2. How determined to follow the diet do you think your child feels? Has their confidence following the diet changed over time?

3. How determined do you feel? Do you both plan to continue the diet for the next 5 years?

4. Would it be true to say that you and your child are now able to follow the diet automatically without thinking at home? How about at school?

5. What are some of the barriers you and he/she are still facing? What has enabled you to succeed?

6. I am circling back to the idea of using medication that we talked about earlier in the interview. At diagnosis you commented you/your family would have felt { } about adding them. Now that you have had more time, if you had had an option to start [*patient*] on a medication to help, would you choose this? The medications I am thinking about are the ones you may have heard in the news called GLP-1 receptor agonists with names like semaglutide, Ozempic, wegozy.
   - Tell me more – open ended

D. Closing (2 min):

1. If you were able to tell your doctors one thing that would help to make adapting to [*rx diet*] easier for you and your child, what would you want us to know?

© 2026 Stephenson KM et al. *JAMA Network Open*.

2. Thank you for all the valuable information that you have shared and your time today. Is there anything else you would like to share before we wrap up?

<u>Surveys and Screens (7 min):</u>
Demographics Survey
Parent Characteristics
Health Literacy Screen

During the interview, you mentioned (*food insecurity, financial insecurity, problems about diet that require clarification*). I would be happy to connect you with our dieticians for a new appointment / our social work team. Would that be helpful?

That concludes the interview. We are all finished. Thank you so much again for sharing your experience and insights with us. This will help us improve how we care for families. I will let the Dr. Stephenson know that we are done, and she will send you your gift card. You should receive it in your email inbox within two days.

Have a nice rest of your day.

© 2026 Stephenson KM et al. *JAMA Network Open*.

Addendum:
Clarifying Probes:
"It sounds like you are saying… is that an accurate summary?"
"When you say, ' ' what do you mean by that?"
"What you're saying now is important and I want to make sure that I understand exactly what you mean. Please explain some more"

Detail Probes:
"Tell me more about that
"Would you elaborate more on that?
"That's helpful, could you provide more detail?"
"What you are sharing is important. Can you say more?"
"How so?"
"Can you give me an example"
"If you could change anything about that experience, what would it be?"
"How do you do that?"
"Can you tell me more about that?"
"Is there anything else?"
"Can you say something about why this issue generated so much emotion?"
"How do you feel about that?"
"Why is that important to you?"
"Why does that stand out in your memory?"
"Why do you think you noticed that?"
"What was significant about this to you?"
"Tell me about the last time this happened?"

Health Literacy Screening Documents:
**https://cdn.mdedge.com/files/s3fs-public/Document/September-2017/026120024.pdf*
**https://pubmed.ncbi.nlm.nih.gov/15343421/*

© 2026 Stephenson KM et al. *JAMA Network Open*.
