# Supplementary material for: Perceptions of GLP-1 RA Use for Children With Obesity Among Caregivers With Food Insecurity: A Qualitative Study
Source: JAMA Netw Open. 2026 Jan 7;9(1):e2552825. doi: 10.1001/jamanetworkopen.2025.52825 (PMC12780928; doi:10.1001/jamanetworkopen.2025.52825)
Supplement: Supplement 2. — Data Sharing Statement [file jamanetwopen-e2552825-s002.pdf]

Data Sharing Statement

Stephenson. Perceptions of GLP-1 RA Use for Children With Obesity Among Caregivers With Food Insecurity. *JAMA Netw Open*. Published January 07, 2026.
doi:10.1001/jamanetworkopen.2025.52825

Data
Data available: No
